# The complete chloroplast genome sequence of *Chimonobambusa luzhiensis*, an endangered species endemic to Guizhou Province, China

**DOI:** 10.1080/23802359.2022.2098856

**Published:** 2022-07-25

**Authors:** Mingli Wu, Yanjiang Liu, Xue Xu, Xiao Zhu, Guangqian Gou, Zhaoxia Dai

**Affiliations:** a College of Life Sciences/Institute of Agro-bioengineering, Key Laboratory of Plant Resource Conservation and Germplasm Innovation in Mountainous Region (Ministry of Education), Guizhou University, Guiyang, China; b Bamboo Research Institute, Guizhou University, Guiyang, China; c College of Forestry, Guizhou University, Guiyang, China

**Keywords:** *Chimonobambusa luzhiensis*, chloroplast genome, phylogeny, phylogenetic analysis

## Abstract

*Chimonobambusa luzhiensis* (Poaceae: Bambusoideae) is an endangered plant endemic to Guizhou Province, China. Here, we report the complete chloroplast genome of *C. luzhiensis*. The plastid genome revealed a typical quadripartite structure with a length of 139,896 bp, including a large single-copy (LSC, 83,191 bp) region, a small single-copy (SSC, 12,811 bp) region, and a pair of inverted repeat (IR) regions (IRa and IRb, 21,797 bp). A total of 131 genes, including 86 protein-coding genes (PCGs), eight ribosomal RNA (rRNA) genes, and 37 transfer RNA (tRNA) genes were annotated, and the overall GC content was 38.8%. Phylogenetic analysis showed that the relationship between *C. luzhiensis* is sister to *C. tumidisinoda*.

The genus *Chimonobambusa* includes 37 species and is widely distributed in southwestern China, Japan, Vietnam, Myanmar, and India (Wang and Hsueh 1993). The stem of *Chimonobambusa* has a square-shaped structure, with bamboo shoots growing from September to November. Shoots of this genus are delicious and considered a high-quality vegetable in China. *Chimonobambusa luzhiensis*, an endemic species from Guizhou, China, is widely distributed in broad-leaved forests at an altitude of 1700–1900 m. In 2013, *C. luzhiensis* was listed as an endangered species in the Redlist of China’s Biodiversity (http://www.iplant.cn/rep/protlist/4). To date, three plastid genomes of *Chimonobambusa* (Liu, Su, et al. 2021; Liu, Zhang, et al. 2021; Zhao et al. 2021; Xu et al. 2022) have been reported: *C. hejiangensis*, *C. sichuanensis*, and *C. angustifolia*. In this study, we reported a new chloroplast genome of this genus (*C. luzhiensis*), which will contribute to the genetic and conservation research of this plant.

Fresh leaves of *C. luzhiensis* were collected from Luzhi county, Guozhou, China (26°17′34″N, 105°18′46″E, 1668 m) in July 2021, and immediately dried with silica. The voucher specimen was deposited in the Natural Museum of Guizhou University (GACP). Total genomic DNA was extracted according to the modified CTAB method (Doyle and Doyle 1987) and sequenced by Illumina NovaSeq 6000 sequencing platform. A total of 4.66 Gb clean reads were obtained after removing low-quality reads and adaptor sequences. The NOVOPlasty4.3.1 software was used to de novo assemble the complete chloroplast genome of *C. luzhiensis* (Dierckxsens et al. 2017) with the *Chimonobambusa hirtinoda* (GenBank accession: MT576658) as a reference. Then, the assembled chloroplast genome sequence was annotated using the PGA (Qu et al. 2019), and manual adjustment was conducted with the Geneious R9 (Kearse et al. 2012). The cp genome sequence of *C. luzhiensis* was submitted to GenBank and the accession number is OM935760.

**Figure 1.**
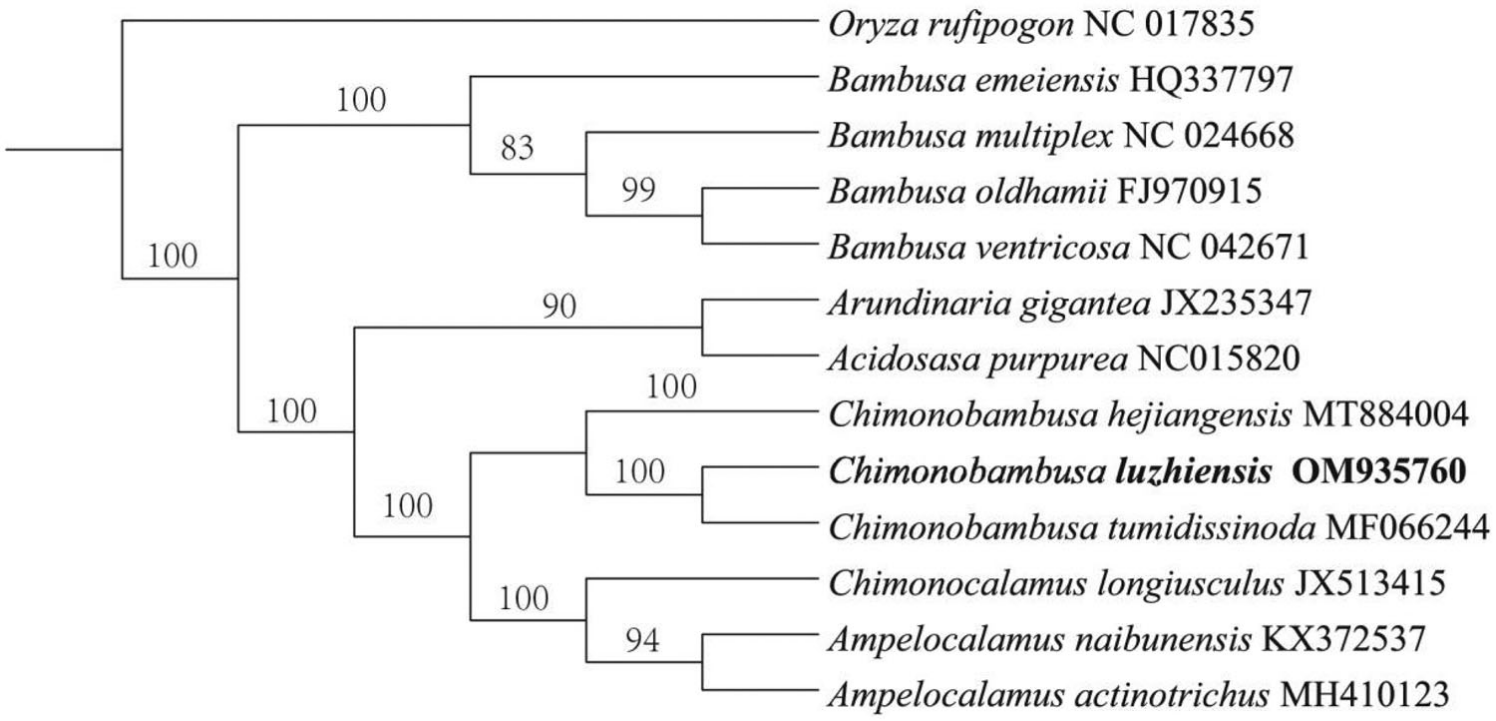
Maximum-likelihood tree of 13 species based on complete chloroplast genomes, with *Oryza rufipogon* as outgroup.

The cp genome of *C. luzhiensis* is 139,596 bp in length and has a typical quadripartite structure. It includes a large single-copy (LSC) region of 83,191 bp, a small single-copy (SSC) region of 12,811 bp, and two inverted repeat regions (IRA and IRB) of the same 21,797 bp. The complete genome GC content was 38.9%, and the corresponding values of the LSC, SSC, and IR were 37.0%, 33.2%, and 44.2%, respectively. It is similar to other bamboo species of the genus *Chimonobambusa*. This chloroplast genome encodes a total of 131 genes, including 86 protein-coding genes (PCGs), 37 transfer RNA (tRNA) genes, and eight ribosomal RNA (rRNA) genes, with 37 duplicated genes (15 PCGs, 14 tRNA genes, and eight rRNA genes).

To further reveal the phylogenetic status of *C. luzhiensis*, based on the whole chloroplast genomes of 13 species of Bambusoideae, a maximum-likelihood phylogenetic tree was constructed, with *Oryza rufipogon* as the outgroup. The 13 complete chloroplast genomes were aligned using online MAFFT 7.0 (Katoh and Standley 2013) and the phylogenetic tree was performed using IQ-tree1.6.12 (Jana et al. 2016). Phylogram showed that the relationship *C. luzhiensis* is sister to *C. tumidisinoda*. The acquisition of the *C. luzhiensis* chloroplast genome will provide useful genetic information for further studies on the genetic diversity and conservation of Bambusoideae (Figure 1).

## Data Availability

The genome sequence data that support the findings of this study are available in GenBank of NCBI under the accession number OM935760. The associated BioProject, SRA, and Bio-Sample numbers are PRJNA814823, SRR18311006, and SAMN26586846, respectively.

## Research license (specimen collection statement)

*Chimonobambusa luzhiensis* (*Chimonobambusa luzhiensis* (Hsueh & T.P.Yi) K.M.Lan 1988) is an endemic bamboo plant in Guizhou Province, China. Due to its important scientific research value and relatively small distribution area, *C. luzhiensis* was listed as an endangered species in the Redlist of China’s Biodiversity. The research team of Professor Gou at Guizhou University intends to study the chloroplast of *C. luzhiensis*. The research team obtained a sampling permit from the Wildlife and Forest Plant Management Station of Guizhou (10 June 2021). The research team complies with the International Union for Conservation of Nature (IUCN) policies research involving species at risk of extinction (see Guidelines for appropriate uses of IUCN Red list data), the Convention on Biological Diversity, and the Convention on the Trade in Endangered Species of Wild Fauna and Flora.
